# Olfaction in Three Genetic and Two MPTP-Induced Parkinson’s Disease Mouse Models

**DOI:** 10.1371/journal.pone.0077509

**Published:** 2013-10-30

**Authors:** Stefan Kurtenbach, Sonja Wewering, Hanns Hatt, Eva M. Neuhaus, Hermann Lübbert

**Affiliations:** 1 Department of Cell Physiology, Ruhr University Bochum, Bochum, Germany; 2 Department of Animal Physiology, Ruhr University Bochum, Bochum, Germany; 3 NeuroScience Research Center, Charité-Universitätsmedizin, Berlin, Germany; 4 Cluster of Excellence NeuroCure, Charite-Universitätsmedizin, Berlin, Germany; University of Sheffield - MRC Centre for Developmental and Biomedical Genetics, United Kingdom

## Abstract

Various genetic or toxin-induced mouse models are frequently used for investigation of early PD pathology. Although olfactory impairment is known to precede motor symptoms by years, it is not known whether it is caused by impairments in the brain, the olfactory epithelium, or both. In this study, we investigated the olfactory function in three genetic Parkinson’s disease (PD) mouse models and mice treated with MPTP intraperitoneally and intranasally. To investigate olfactory function, we performed electro-olfactogram recordings (EOGs) and an olfactory behavior test (cookie-finding test). We show that neither a parkin knockout mouse strain, nor intraperitoneal MPTP treated animals display any olfactory impairment in EOG recordings and the applied behavior test. We also found no difference in the responses of the olfactory epithelium to odorants in a mouse strain over-expressing doubly mutated α-synuclein, while this mouse strain was not suitable to test olfaction in a cookie-finding test as it displays a mobility impairment. A transgenic mouse expressing mutated α-synuclein in dopaminergic neurons performed equal to control animals in the cookie-finding test. Further we show that intranasal MPTP application can cause functional damage of the olfactory epithelium.

## Introduction

Parkinson’s disease (PD) is the second most common neurodegenerative disorder after Alzheimer’s disease and the most common disorder with motor deficits due to the degeneration of dopaminergic neurons in the substantia nigra [1]. While most cases are sporadic, 5–10% of the patients suffer from inherited PD, which can be associated with mutations in 11 genes to date, among them α-synuclein (α-syn) and parkin [2]–[6]. The etiology of PD remains poorly understood, but it is postulated that a combination of genetic predispositions and environmental toxins increase the risk for PD [5].

Although PD is considered to be a motor-system disease, non-motor symptoms often precede the motor symptoms, which are caused by damage in different brain areas. Autopsy-based studies by Braak and coworkers demonstrate a caudal to rostral disease progression in six phases (Braak stages) from the Meissner and Auerbach’s plexuses, vagus and glossopharyngeal nerves, spinal cord towards the nucleus of the vagus nerve and the substantia nigra [7]. In some cases the pathology spreads further to mesocortical and neocortical areas. The six phases are characterized by the appearance of Lewy neurites and Lewy bodies in the different regions which mostly consist of aggregated α-syn, where unmyelinated neurons are more vulnerable to degeneration [8], [9]. Already at very early stages of PD, 70–95% of the patients exhibit deficits in odor detection and discrimination [10]–[12]. These olfactory deficits often precede motor symptoms by up to four years [13]–[17]. Of the six so-called Braak stages the Braak1 stage is already characterized by pathological changes in the olfactory bulb and anterior olfactory nucleus, maybe causing the patients’ olfactory deficits. Olfactory perception may therefore serve as a tool for early diagnosis.

Toxic and transgenic mouse models have been used to elucidate the molecular mechanisms of PD pathogenesis [18]. The neurotoxin 1-methyl-4-phenyl-1,2,3,6-tetrahydropyridine (MPTP) is well known to produce features in mice, monkeys and humans that are strikingly similar to PD [19], such as a reduction in the density of dopaminergic axons in the striatum and dopaminergic cell bodies in the substantia nigra compacta [20]. Intragastrically administered rotenone was shown to induce α-synuclein accumulation and reproduce PD pathological staging as found in humans [21]. The Olfactory epithelium can serve as a route for neurotoxins to the brain, as mice can develop memory and sensory deficits after a single intranasal MPTP injection [22], [23], causing reduced tyrosine hydroxylase (TH) levels in the olfactory bulb, substantia nigra, and the striatum.

Transgenic mouse lines carrying PD-inducing gene mutations have been characterized as genetic models for PD [24]. We have previously generated mice overexpressing human doubly mutated (A30P, A53T) α-synuclein under the control of the β-actin (BAsyn) or the tyrosine hydroxylase promoter (THsyn) [25] and knockout mice lacking parkin (PaKO). None of these transgenic mouse lines show severe histopathological alterations in the substantia nigra. However, they display damaged mitochondria in neurons and glial cells [26], [27]. BAsyn mice exhibit neuronal cell death in the spinal cord, accompanied by extensive gliosis and microglial activation [28], in accordance with the staging scheme of PD-patients proposed by Braak and coworkers [7]. Testing whether these mouse models also exhibit deficits in olfactory perception or discrimination might provide further information on the reflection of early PD symptoms in the model. Moreover, since the causes of most PD cases are multifactorial, we investigated the effect of MPTP on the olfactory system of the transgenic mice to elucidate if the combination of genetic and toxic predispositions increases a PD-related pathology in the mouse models.

## Results

### Olfactory Performance of Transgenic PD Mouse Lines

We tested three different transgenic mouse lines resembling genetic modifications associated with PD. BAsyn mice express mutated human α-synuclein under the control of the ubiquitous β-actin promoter [25], [27]. These mice exhibit severe histopathological changes in the spinal cord, potentially indicative of an early PD-related pathology. We also investigated two other mouse lines, one overexpressing mutated human α-synuclein in dopaminergic neurons (THsyn), and the other one lacking parkin (PaKO).

**Figure 1:**
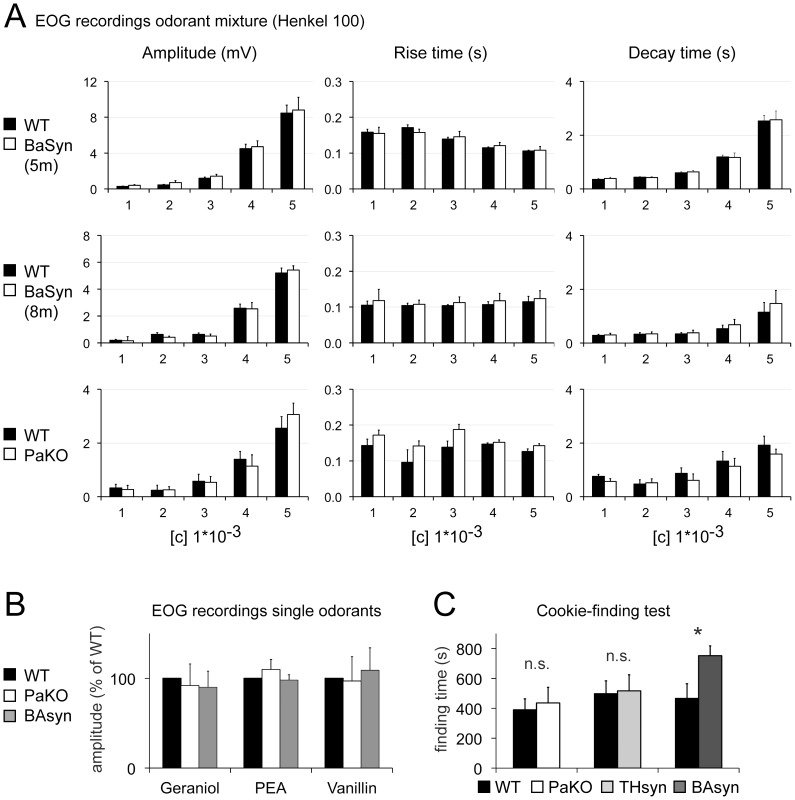
EOG recordings and cookie-finding tests of different mouse strains. (**A**) EOG recordings from PaKO and BAsyn mice at 5 months (5 m) and 8 months (8 m) of age. Black and white bars represent the mouse lines listed on the left. n_Parkin_ = 6, n_BAsyn5m_ = 5, n_BAsyn8m_ = 6, n_controls_ = n_transgenic_. Five different dilutions [c] of Henkel 100 were applied (100 ms duration). No significant differences could be detected. (**B**) Normalized EOG recordings (n = 4) after single odorant applications (geraniol 1∶10, vanillin 40 mM, phenylethylamine (PEA) 1∶1000). Error bars represent SEM. (**C**) Performances of different mouse lines in the cookie-finding test. Data was normalized to wild type animals and depicts latency to find the cookie. BAsyn mice (8 m) took significantly longer to find the cookie. n_Parkin_ = 10 (p = 0.75), n_BAsyn_ = 12 (p = 0.04), n_ThSyn_ = 10 (p = 0.91), n_controls_ = n_transgenic_. Error bars represent SEM.

To investigate the functionality of the olfactory epithelium (OE), we performed electro-olfactogram (EOG) recordings. A mixture composed of 100 different odorants (Henkel 100) was applied via a constant, humidified airstream to activate a large fraction of olfactory receptor neurons. Five concentrations with odorant dilutions ranging from 1∶10,000,000 to 1∶1,000 were tested. The results for the EOG amplitude, rise time and decay time are depicted in Figure 1A. Because pathological symptoms increase in BAsyn mice with age, we examined animals with beginning symptoms (5 months) and animals with pronounced symptoms (8 months). 8 months is the maximum age where these mice can be used for testing, since they die shortly after this age. Neither BAsyn nor PaKO mice (lower panels) showed differences to WT animals in EOG amplitudes and response kinetics (Figure 1A). We further applied three single odorants, geraniol (1∶10), phenylethylamine (PEA, 1∶1000) and vanillin (40 mM) and also find no significant differences in the EOG recordings (Figure 1B). These results indicate that the transgenic mice investigated do not display an impaired OE function. To test olfactory perception in general, we additionally performed cookie-finding tests (Figure 1C), in which the latency to find a cookie hidden in the home cage of the mice was determined. Animals were trained the day before testing with a 500 mg cookie, rendering the test very easy, to familiarize the animals with the cookie scent. After the training day, we used 50 mg cookies, provide only a very weak stimulus, which in our experience renders the test difficult. PaKO and THsyn mice did not perform significantly different from their wild type littermates (Figure 1C). BAsyn mice took significantly longer to find the cookie, but displayed a reduced mobility compared to wild type mice (see below).

### Effect of Intraperitoneal MPTP Administration on Olfactory Perception

**Figure 2:**
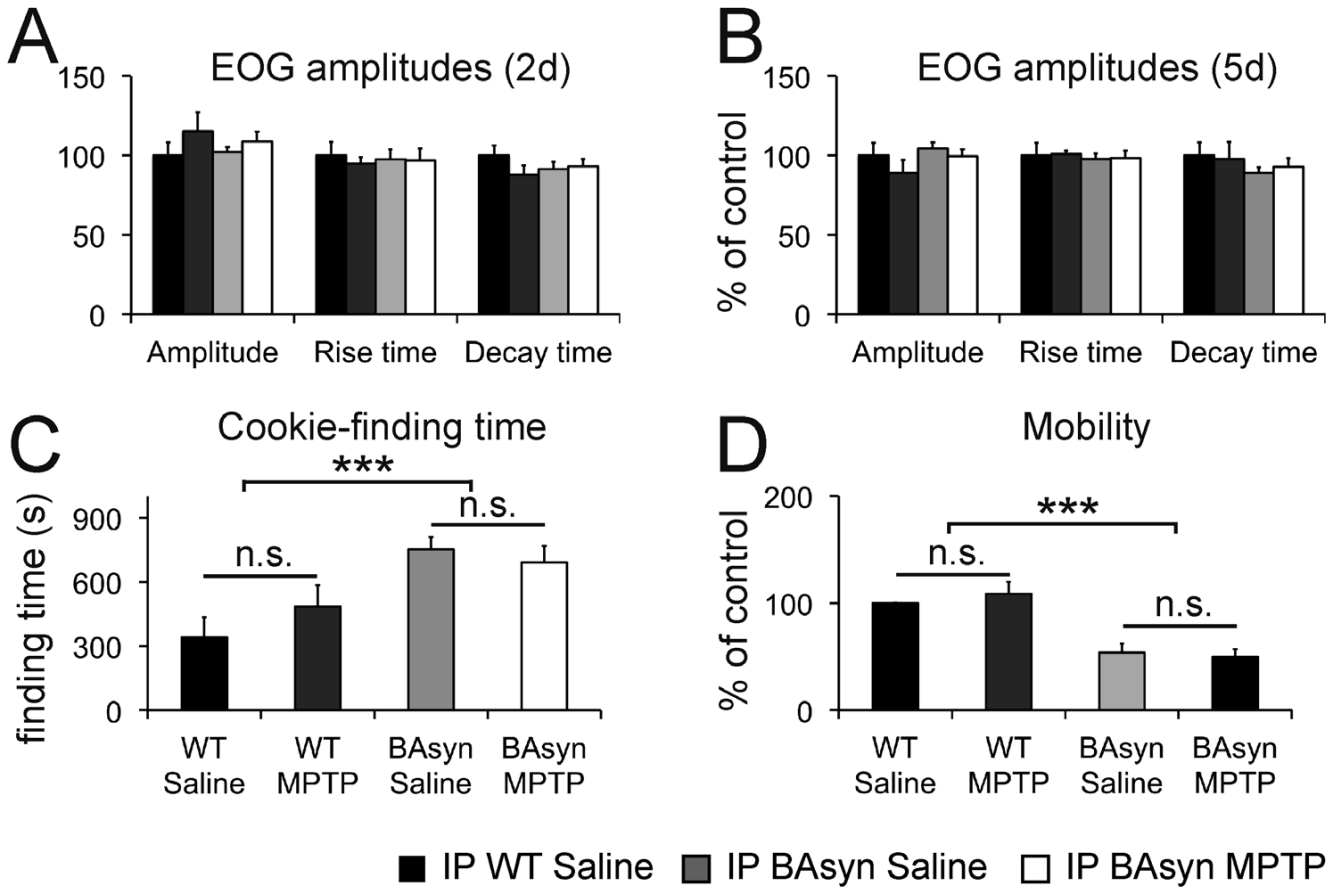
EOG recordings and cookie-finding tests of intraperitoneally (IP) MPTP injected animals. EOG data from mice injected with MPTP intraperitoneally (**A**) two days (2d) and (**B**) five days (5d) after treatment. The amplitudes of the responses, the rise, and the decay times did not differ significantly between the groups. n_IP_ = 5 animals per condition. (**C**) Cookie-finding tests after 5 days of IP MPTP injection showed no significant difference between saline and MPTP treated animals (n = 10 per condition). BAsyn animals took significantly longer to find the cookie. (**D**) Animal mobility (mean movement velocity) during the cookie-finding test. BAsyn animals display a significantly reduced mobility. Error bars represent SEM.

In addition to the transgenic models, we also used MPTP to induce a PD like phenotype. Mice were injected intraperitoneally with 15 mg MPTP/kg body weight, twice per day at intervals of 2 h, on two consecutive days. Intraperitoneal injection of MPTP is an established method to induce PD-like pathogenic symptoms in the mouse brain [20]. The results of the EOG recordings in this mouse models are shown in Figure 2A and B. The responses of the OE to odorants were not affected by intraperitoneal MPTP application, neither at two, nor at five days after the injections. The amplitudes and the kinetics of the recorded responses also did not differ significantly between saline and MPTP treated WT animals. In addition, intraperitoneal injections of MPTP did not lead to a significant increase in the cookie-finding time 5 days after treatment in WT mice, as depicted in Figure 2C, and not affected the mobility of the animals (Figure 2D). In a later experiment, we could nevertheless see a significantly reduced number of striatal dopaminergic fibers after MPTP application, proving that the MPTP injections were effective (see below). Since PD is thought to be caused by a combination of genetic predispositions and environmental effects, we also tested whether transgenic mice with genetic changes common in PD may be more susceptible to neurotoxins than wild type mice. MPTP was thus injected intraperitoneally to BAsyn mice (8 months old), as these show the strongest pathological phenotype of our mouse strains. Intraperitoneal injection of MPTP to BAsyn mice did not result in differences in the EOG responses compared to wild type or saline-treated BAsyn mice (Figure 2A, B). MPTP treatment also did not increase the cookie-finding time and the mobility compared to saline treated BAsyn mice (Figure 2C, D).

### Effect of Intranasal MPTP Administration on Olfactory Perception

**Figure 3:**
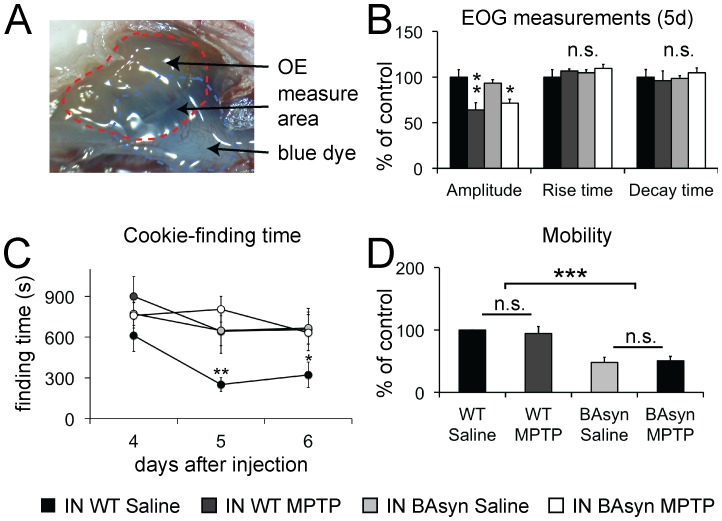
EOG recordings and cookie-finding tests after intranasal (IN) MPTP application. (**A**) Olfactory epithelium after intranasal application of 5 µl blue dye. EOGs were measured in the indicated areas of the OE. (**B**) EOG data from mice treated wit 0.5 mg/nostril MPTP intranasally. The amplitude was significantly reduced in MPTP treated wildtype (p<0.01), and BAsyn animals (p<0.05), while the rise and the decay time of the responses did not differ (n_MPTP_ = 6, n_saline_ = 10). (**C**) After the training day (day 4), wildtype mice treated IN with MPTP treated took significantly longer to find the cookie 5 days (p<0.01) and 6 days (p<0.05) after MPTP application (n = 10 for each condition), compared to control animals. Finding time of BAsyn mice was not increased after IN MPTP treatment. (**D**) Animal mobility (mean movement velocity) during the cookie-finding test. Data is normalized to the wild type animals. No significant difference was detectable between saline and MPTP treated animals. BAsyn mice display a significantly reduced mobility (p<0.01). Error bars represent SEM.

We further applied MPTP intranasally, a method discussed to elicit olfactory deficits resembling those in preclinical PD [23]. We applied 0.5 mg MPTP per nostril in a volume of 5 µl. To check which areas of the OE were in direct contact with the applied liquid, we injected 5 µl of a coomassie dye solution into the nasal cavity (Figure 3A). EOGs were then measured only in the area of the OE that was stained blue in this initial experiment. Before performing EOG measurements, we checked the OE of intranasally injected animals thoroughly, but could not detect any signs of lesions, bleedings or other abnormalities. Figure 3B illustrates the data obtained from EOG measurements. Intranasal administration of MPTP decreased the EOG amplitudes significantly in both, WT and BAsyn mice, whereas the kinetics of the responses did not differ. The latency to find the cookie in the cookie-finding test was significantly increased in WT but not BAsyn animals after intranasal MPTP application, on two consecutive test trials (day 5 and 6, the training day showed not significant differences. Figure 3C). We found no significant changes in the animals’ mobility after intranasal MPTP application (Figure 3D). The mobility of BAsyn mice was significantly lower than that of WT animals.

### Immunohistochemical Analysis of Olfactory Bulb and Striatum

**Figure 4:**
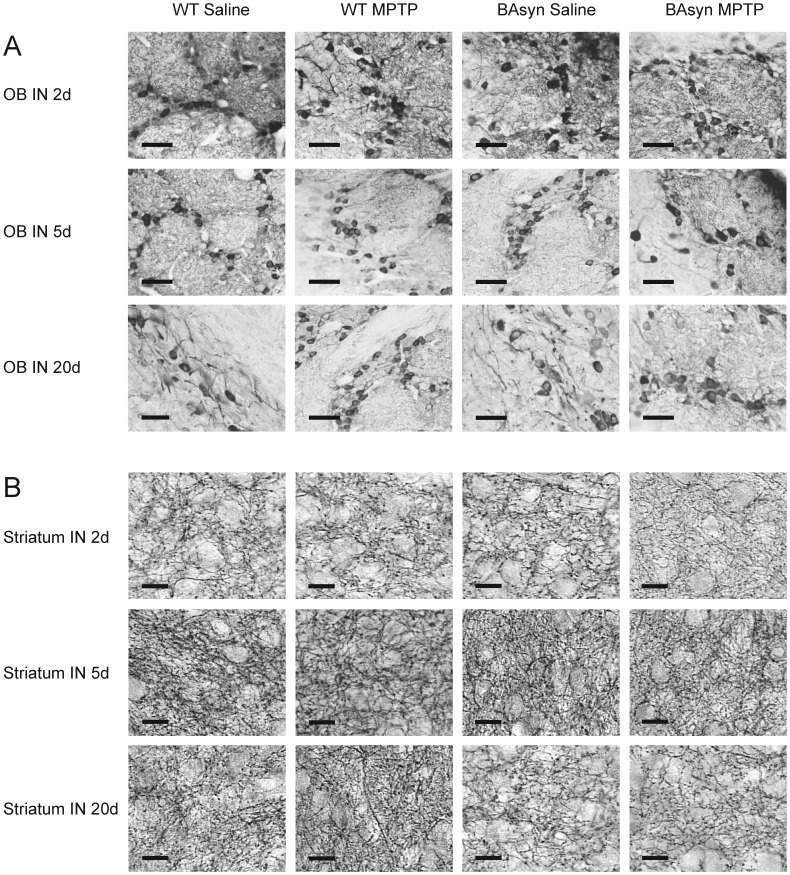
TH immunostainings of the olfactory bulb and striatum after intranasal MPTP administration. Light microscopic analysis after anti-tyrosine hydroxylase (TH) staining of the olfactory bulb (OB) and the striatum of BAsyn and wild type mice. Mice were investigated 2, 5 or 20 days (d) after intranasal (IN) MPTP treatment with 0.5 mg/nostril. Scale bars: 50 µm. Pictures are representatives of three biological replicates.

**Figure 5:**
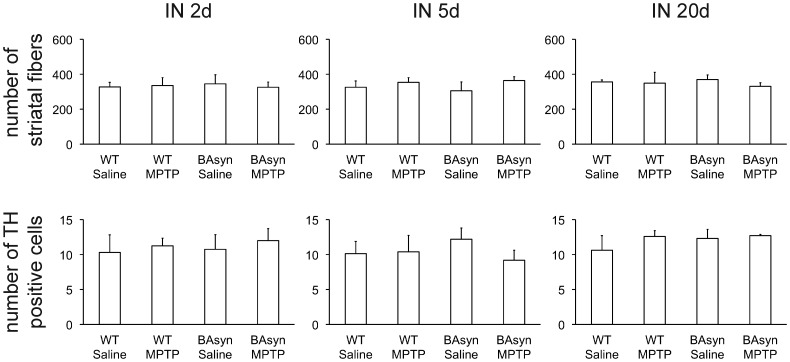
Quantification of the light microscopic analyzes after anti-tyrosine hydroxylase (TH) staining of the olfactory bulb (OB) and the striatal fibers. No significant differences were seen 2, 5 and 20 days after intranasal application of 0.5(p>0.05, n = 3, striatal fibers were analyzed in a field of 45×45 µm, TH positive neurons were manually counted in the glomerular layer of the OB (total magnification 200×)). Error bars represent SEM.

**Figure 6:**
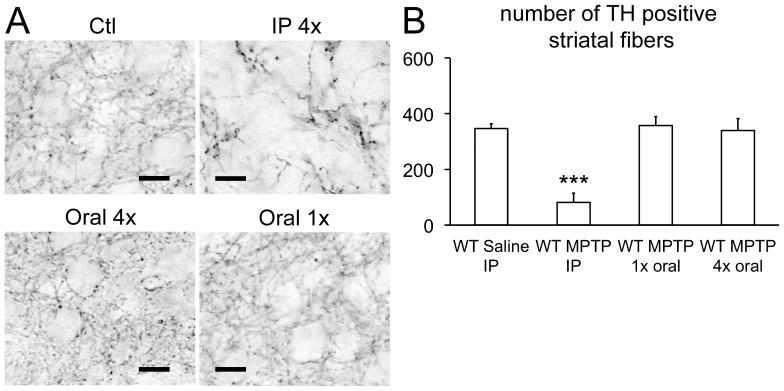
Histopathological alterations can be seen after IP MPTP injection, but not oral application. (**A**) Representative pictures of the striatum after anti-tyrosine hydroxylase (TH) staining and quantification (**B**). Mice were investigated two days after MPTP treatment. Reduction of TH positive fibers is only seen in IP treated animals (p<0.001). Animals were either treated four times (4×) with 15 mg/kg MPTP (see Methods) or with one (1×) single dose of 60 mg/kg. Scale bars: 50 µm. n = 3, striatal fibers were analyzed in a field of 45×45 µm. Error bars represent SEM.

To analyze the histopathological changes caused by intranasal application of MPTP, we investigated the olfactory bulb (OB) and striatum of BAsyn and wild type mice 2, 5 and 20 days after intranasal injection of MPTP. Staining was performed with an antibody against tyrosine hydroxylase (TH), to visualize dopaminergic neurons. Representative stainings are shown in Figure 4, quantification of the data in Figure 5. The numbers of TH positive cells in olfactory bulbs of animals treated with MPTP intranasally does not differ significantly from the respective controls, and also the axon density in the striatum is normal, as revealed by quantification of 3 animals each (Figure 5). In case of intraperitoneal MPTP injection we found a prominent reduction of TH positive fibers in the striatum, as depicted in Figure 6, which is in line with previous work [20]. We further tested if intranasal application of MPTP in higher volumes may cause a reduction in TH-positive fibers in the brain because a significant amount of liquid is swallowed by the animals and reaches the brain via the gastrointestinal tract. To test this, we applied MPTP orally. One mouse group received 15 mg/kg MPTP on two consecutive days, two oral injections per day, at 2 h interval for a cumulative dose of 60 mg/kg. A second group received a single dose of 60 mg/kg. Figure 6 shows that oral application of MPTP did not lead to changes in the density of TH-positive fibers in the striatum.

## Discussion

Impaired olfactory capability has been reported to accompany various neurodegenerative diseases [29]–[32] and may serve as pre-symptomatic disease indicator. It is not known, whether the olfactory impairments in PD are caused by impairments in the brain, the OE or both. Since signs of pathology can be found in the olfactory bulb at early stages [7], the impairments may be caused by beginning brain damage. In this study we investigated transgenic and toxin induced mouse models resembling some aspects of early stage PD pathology, to elucidate whether olfactory deficits are present and whether they originate in the OE or the brain.

### Transgenic Mice

We investigated the olfactory system of three transgenic mouse lines resembling genetic modifications known to be involved in disease formation in humans. α-Synuclein expression is driven by the β-actin promoter in BAsyn mice, and by the tyrosine hydroxylase (TH) promoter in THsyn mice. The third mouse line (PaKO) lacks the expression of parkin, an ubiquitin ligase mutated in some cases of juvenile Parkinsonism. Our EOG recordings show that the olfactory epithelium of BAsyn, THsyn and PaKO mice functions normally, as EOG amplitudes and response kinetics differed not significantly compared to control animals. To prove that our EOG measurements are sensitive, we measured NKCC1^−/−^ mice, a mouseline reported to display reduced EOG responses in two studies (−39% and −54%) [33], [34]. We find a reduction of EOG amplitudes in NKCC1^−/−^ mice of −71% (Figure S1, n_KO_ = 5, n_WT_ = 5), proving our method to be highly sensitive (Figure S1). The even higher effect compared to the previous studies may be explained by the difference in odorant concentration, use of odorant mixture, application procedure and/or measurement setup. We further used a cookie-finding paradigm to test for olfactory deficits in general. We found that THsyn and PaKO mice perform equally as wildtype animals at this test, implicating that they do not have a major olfactory impairment, although it must be noted that the cookie-finding test can only cover for certain aspects of olfaction and it may still be possible that the animals have a subtler or specific olfactory impairment that was not resolved by this test. BAsyn mice took significantly longer to find the cookie, but since their mobility turned out to be significantly decreased, the result cannot be interpreted.

### The MPTP Model

Intraperitoneally applied MPTP causes a strong reduction of TH-positive fibers in the striatum (Figure 6), which is in accordance to previous work [20]. We find no differences in the numbers of TH positive cells in the olfactory bulb. We also find no changes in EOG recordings, showing that the function of the OE is not impaired by intraperitoneal MPTP injection. Cookie-finding tests showed that the animals do not have a major olfactory impairment since the performance of MPTP treated animals was similar to control animals. This finding gains support from the fact that humans with MPTP induced Parkinsonism do not have olfactory impairments, in contrast to patients with idiopathic PD [35].

Mice treated with MPTP intranasally (0.5 mg/nostril) displayed an increased cookie-finding time. We did not find any differences in the number of dopaminergic fibers in the striatum and the number of TH positive cells in the olfactory bulb, in contrast to other studies, which though used higher amounts of MPTP [23] (1 mg/nostril). We do find an impairment of the OE, as EOG response amplitudes were significantly decreased in BAsyn and WT animals after intranasal MPTP application. This finding raises the possibility that the differences in olfactory behavior tests after intranasal application of MPTP are caused by OE damage. MPTP itself is not toxic to neurons, but can pass the blood brain barrier. Glial cells express monoaminooxidase B (MAO-B), which converts MPTP to the dopaminergic neurotoxin 1-methyl-4-phenylpyridinium (MPP+). MPP+ damages the mitochondrial respiratory chain by interfering with complex I (for review see [36]). The OE expresses MAO-B [37], which may convert MPTP to MPP+ to some extent, damaging the OE and leading to the observed reduction of the EOG amplitudes. The fact that severe damage in the brain was only seen after intraperitoneal, but not intranasal application, may therefore be caused by efficient conversion of MPTP to MPP+ in the OE. However, it still is possible that brain damage has occurred and contributes to the olfactory impairment because first, MPTP toxicity could have resulted in other effects than changing TH immunreactivity and second, our immunhistochemical analysis may not be sufficient to unravel less prominent changes (n = 3, mixed sex). Generally, MPTP is age and strain dependent [38]–[40], explaining why a single intranasal application may be used to elicit histopathologic changes in the brain, possibly resembling preclinical olfactory loss present in PD [23], but in other cases is reported to have less severe effects [41]. We further show that oral application of MPTP does not lead to a reduced number of TH-positive fibers in the striatum and the OB (Figure 6), which is in agreement with the finding that after oral application of MPTP no MPP+ is found in the brain [42]. This excludes the possibility that MPTP, when applied in higher volumes, can reach the brain over the gastrointestinal tract. BAsyn mice treated with MPTP intranasally did not show an increased cookie-finding time, likely caused by their motoric deficits mask a potential effect of the olfactory impairment. Other olfactory behavior tests, relying on the animals’ mobility to a lower extent, could be used to resolve this question.

In recent years, a huge variety of PD animal models were generated, some of which were reported to develop more pronounced olfactory impairments with age, correlating with the fact that PD develops mainly in older people. For instance, a recent study using a double transgenic animal reports an olfactory impairment more pronounced in animals that are one year old, in comparison to animals that are 8 months old [43]. Also dopamine deficient transgenic animals, for instance VMAT2-deficient mice, demonstrate progressive effects in odorant discrimination [44]. Synuclein pathology in olfactory projections was also shown to correlate and progress during aging in mice [45]. Thy1-asyn mice were reported to have an olfactory deficit in behavior tests, also in a cookie-finding test [46]. These studies indicate, that genetic and toxin induced models of PD can possess olfactory impairments. It is important to note that genetic and toxin induced models mimic only certain aspects of PD, and both have limitations regarding comparison to human PD. Genetic overexpression of BAsyn does not correlate with the temporal stages described in humans. MPTP on the other hand mainly induces dopaminergic and motoric dysfunctions of the disease. Still it is important to understand if and how different genetic/toxin-induced models and resulting pathologic pathways lead to an olfactory impairment in different animal models, and where they occur (OE and/or brain) to increase understanding of PD pathology.

In summary, we conclude that higher concentrations of MPTP applied to the OE in larger volumes may be used to study central impairments leading to olfactory deficits, as previously demonstrated [23], but since MPTP damaged the OE in our study, the damage to the OE should be assessed before subjecting the animals to behavior tests. Other application procedures [23], [41] may cause less damage, and as the OE replaces neurons continuously, behavior tests following intranasal MPTP application may still be possible after regeneration. The responsiveness of the OE to odorant application is not altered in PaKO, THsyn, and BAsyn animals, showing that the genetic changes present in these animals do not affect olfactory epithelium function. PaKO and THsyn animals also perform normally in a cookie-finding test. BAsyn animals are not suitable for this test as their mobility is decreased. IP MPTP application does not cause significant changes in OE function and performance in cookie-finding tests, despite a major reduction of TH positive fibers in the striatum present in treated animals.

## Materials and Methods

### Transgenic and Knockout Mouse Lines

We analyzed two α-syn transgenic mouse lines expressing human doubly mutated (A30P and A53T) α-syn in a C57BL/6J background. The transgenes are driven by either (i) the chicken beta-actin promoter (BAsyn) or (ii) the tyrosine hydroxylase promoter (THsyn). We further used a homozygous parkin-deficient line with a targeted deletion of exon3 (PaKO), which was backcrossed with wild-type C57BL/6J (F10-generation; >99% C57BL/6J and <1% 129SvJ). NKCC1 knockout mice (NKCC1^−/−^) were generated by Prof. Dr. Gary E. Shull, University of Cincinnati [47] and kindly provided by Prof. Dr. med. Ursula Seidler, University of Hannover. As controls we used age-matched non-transgenic littermates. Behavioral, histopathological and functional analyses of these lines have been described earlier [25], [27], [28]. Housing and breeding of the animals was performed in accordance with the German guidelines of the animal care and use committee. All efforts were made to minimize the number of animals used and their suffering. If not stated otherwise, we used 8 month old animals, and groups containing male and female mice, were used. The data from male and female mice were combined, as we found no differences in the presented and previous tests.

### MPTP Administration

All animal experiments were in accordance with the German guidelines for animal care and use and were approved by the regional authority (LANUV NRW) and an ethics committee.

### Intraperitoneal Injections of MPTP

Intraperitoneal injections were performed as described previously [20]. Transgenic and wild type animals were treated with MPTP (Sigma, Munich, Germany) dissolved in sterile saline 15 mg MPTP/kg body weight, in sterile physiological saline, twice daily with a 2 h interval on two consecutive days. Control animal received the same volume of saline. Animals were sacrificed by cervical dislocation 2, 5 or 20 days after the last injection.

### Intranasal Injections of MPTP

Mice were anesthetized with 85 mg/kg ketamine and 10 mg/ml xylazine. 0.5 mg MPTP per nostril dissolved in 5 µl sterile saline was injected into each nostril. Injection was performed with Eppendorf GS10 pipette tips.

### Tissue Preparation

Mice were anesthetized with a ketamine (100 mg/kg) and xylazine (10 mg/kg) mixture and perfused transcardially with phosphate buffered saline, followed by 4% paraformaldehyde in 0.1 M phosphate buffer.

### Immunohistochemistry

For immunohistochemistry brains were embedded in paraffin. Sections of 18 µm were deparaffinized and antigen retrieval (5–10 min cooking in 0.01 M citrate buffer, pH 6.0) was performed. After pre-incubation (3% normal goat serum) adjacent sections were treated with a sheep anti-rat tyrosine hydroxylase antibody, serving as a marker for catecholaminergic neurons (anti-TH; 1∶500; Millipore, Schwalbach, Germany). Sections were treated by the corresponding biotinylated secondary antibody (1∶300, Axxora, Lörrach, Germany), ABC reagent (1∶100; Axxora, Lörrach, Germany) and silver-gold intensification [48]. Quantification of axons was performed as described previously [20]. TH positive cells in the glomerular layer of the olfactory bulb were quantified manually by light microscopy (200×magnification). All immunohistochemical analyses were performed on sagittal brain sections at Lateral 1.44 mm (OB 5/4, ST 1/3) according to the Mouse Brain Atlas (Franklin *et al.*, The Mouse Brain in Stereotaxic Coordinates, Third Edition).

### Behavioral Analysis

#### Cookie-finding test

Mice were trained one day to find a cookie (Leibniz Butterkeks; Bahlsen, Hannover, Germany) buried beneath 6 cm of woodchip bedding in their home cage (500 mg). At the following day (testing day) a cookie of 50 mg was hidden. The latency to locate the cookie was recorded. We defined finding the cookie as when the mouse held it in both fore paws.

If a mouse did not find the cookie within 15 minutes, the test was aborted. Cookie-finding tests were analyzed using the u-test, because of data truncation. *p<0.05, **p<0.01, ***p<0.005.

#### Mobility analysis

Animal mobility was analyzed with the EthoVision XT7 Software from Noldus (Wageningen, Netherland). Mobility was defined as distance per time. Tracking was stopped when the animal found the cookie. To test for statistical significance student’s T-test was used.

#### EOG recordings

The skull was cut parasagittal to the septum. The turbinates were removed and EOGs were recorded from the olfactory epithelium (OE) on the septum. A constant humidified air stream was delivered to the OE (2.4 l/min). Odors were injected into the air stream via a custom made device. Odorant pulses had 100 ms duration. Henkel 100 (Henkel, Düsseldorf, Germany), a mixture of 100 different odors [49], was used as a stimulus. Single odorants (geraniol, vanillin and phenylethylamine) were purchased from SIGMA, Schnelldorf, Germany. Dilutions were made in distillated water. Student’s t-test was used to analyze statistical significant differences between the animals, and multiple responses (<10) were recorded and averaged for each animal.

## Supporting Information

Figure S1 EOG recordings from NKCC1^−/−^ mice. Amplitudes of NKCC1^−/−^ mice are significantly reduced (p<0.001) by 71%, whereas the response kinetics, rise and decay time, is do not differ significantly. Error bars represent SEM.
(TIF)
