# Supplementary figures and images for: Olfaction in Three Genetic and Two MPTP-Induced Parkinson’s Disease Mouse Models
Source: PLoS One. 2013 Oct 30;8(10):e77509. doi: 10.1371/journal.pone.0077509 (PMC3813626; doi:10.1371/journal.pone.0077509)

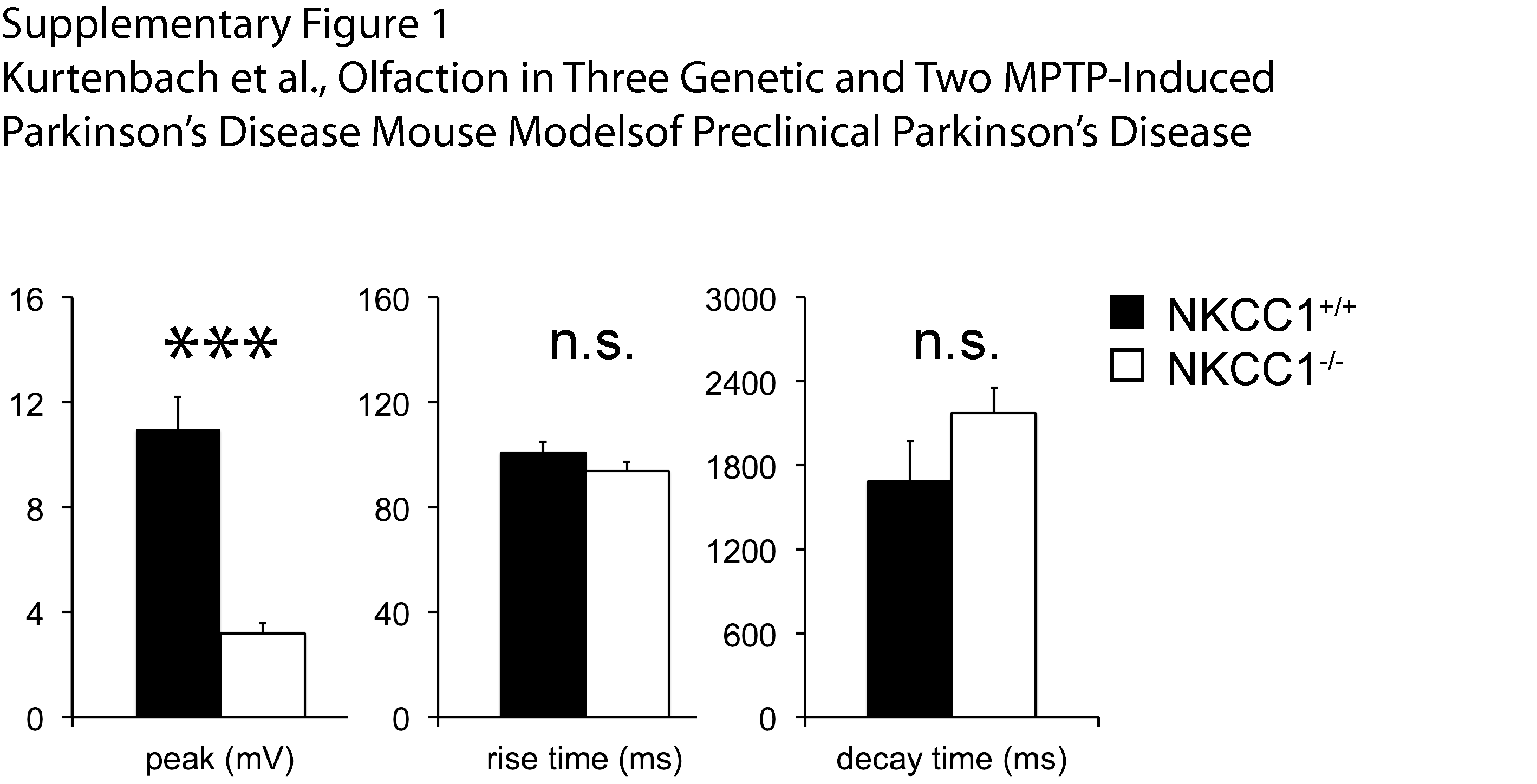

Supplement: Figure S1 — EOG recordings from NKCC1−/− mice. Amplitudes of NKCC1−/− mice are significantly reduced (p<0.001) by 71%, whereas the response kinetics, rise and decay time, is do not differ significantly. Error bars represent SEM. (TIF) [file pone.0077509.s001.tif]
